# A Case of Biliary Intraepithelial Neoplasm in a Young Man Diagnosed by Laparoscopic Hepatectomy to Treat Recurrent Intrahepatic Lithiasis and Cholangitis

**DOI:** 10.70352/scrj.cr.24-0048

**Published:** 2025-02-20

**Authors:** Yuto Yamahata, Jungo Yasuda, Hironori Shiozaki, Yasuro Futagawa, Tomoyoshi Okamoto, Toru Ikegami

**Affiliations:** 1 Department of Surgery, Jikei University Daisan Hospital, Komae, Tokyo, Japan; 2 Division of Hepatobiliary and Pancreas Surgery, Department of Surgery, The Jikei University School of Medicine, Tokyo, Japan

**Keywords:** biliary intraepithelial neoplasia, intraductal papillary neoplasm of the bile duct, hepatic resection, intrahepatic lithiasis, cholangitis

## Abstract

**INTRODUCTION:**

Biliary intraepithelial neoplasia (BilIN) is defined as a bile duct epithelial tumor with intraductal papillary neoplasia of the bile duct. BiIlN is a precancerous lesion of intrabiliary neoplasia. We performed laparoscopic hepatic resection for recurrent cholangitis due to intrahepatic lithiasis and diagnosed BilIN. This case suggests that it is necessary to consider the possibility of malignancy in cases of repeat cholangitis due to intrahepatic lithiasis.

**CASE PRESENTATION:**

A 34-year-old man developed cholecystitis due to gallstones at the age of 25 years and underwent laparoscopic cholecystectomy at the age of 26 years. One year later, cholangitis developed, and 2 years later, acute pancreatitis developed due to bile duct stones. Three years later, at the age of 31 years, he underwent endoscopic lithotripsy for bile duct stones and cholangitis. At that time, intrahepatic lithiasis was also detected in segment 6, but there was no stricture in the bile duct, and he was kept under observation. Three years later, at the age of 34 years, cholangitis in the bile duct of segment 6 was observed, and endoscopic nasobiliary drainage was performed. At that time, no strictures or common bile duct stones were found in bile duct of segment 6; however, we decided to perform laparoscopic hepatic resection of the ventral region of segment 6 because of the recurrent cholangitis. Pathological examination revealed bile duct inflammation and BilIN-1 in the bile duct epithelium; the bile duct stump was negative.

**CONCLUSIONS:**

We experienced a case of a young patient with recurrent cholangitis due to intrahepatic lithiasis and diagnosed BilIN after laparoscopic hepatectomy. In such a case, it is also necessary to select a strategy that considers the coexistence of precancerous lesions, such as BilIN.

## Abbreviations

BilIN
: biliary intraepithelial neoplasia
B6
: bile duct of segment 6
S6
: segment 6

## INTRODUCTION

Biliary intraepithelial neoplasia (BilIN) is defined as a biliary epithelial tumor with intraductal papillary neoplasia.^1)^ BilIN is a precancerous lesion of intraductal neoplasia.^2,3)^ BilIN is classified by grade and is thought to progress to high grade due to chronic inflammatory stimulation and develop into cholangiocarcinoma.^4)^ BilIN induces no specific clinical symptoms or imaging findings and, in many cases, is diagnosed by microscopic examination at the time of surgical resection of a cholangiocarcinoma. We report a case of BilIN in which a diagnosis was confirmed by pathological examination after laparoscopic hepatectomy for intrahepatic lithiasis with recurrent cholangitis.

## CASE PRESENTATION

**Fig. 1:**
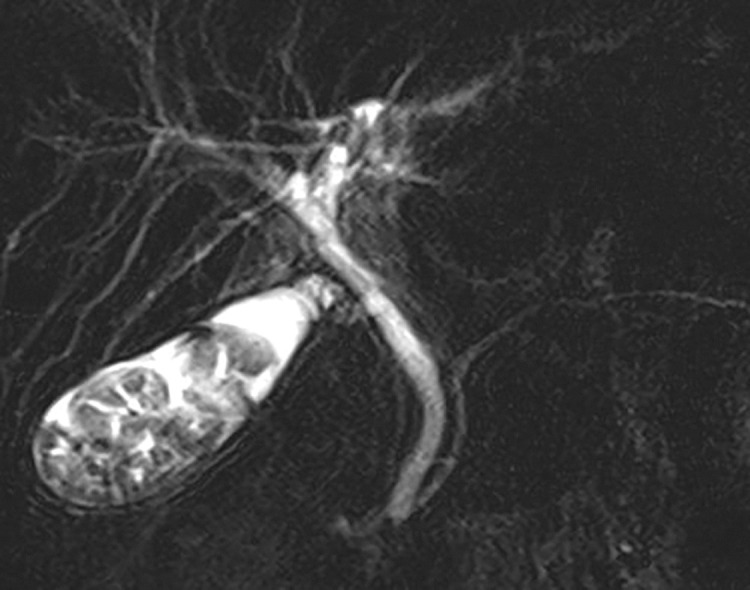
Magnetic resonance cholangiopancreatography. Biliary tract evaluation before cholecystectomy revealed no stricture or abnormality. There were no findings in the intrahepatic bile duct. However, many stones were found in the gallbladder.

**Fig. 2:**
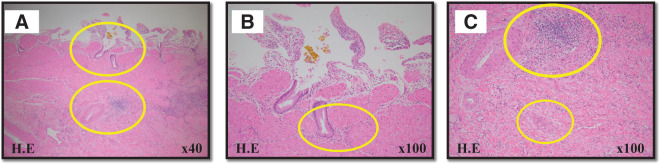
Microscopic images. (**A**) (original magnification ×40) and (**B** and **C**) (original magnification ×100) are hematoxylin-eosin-staining images of the gallbladder. Inflammatory cell infiltration was observed in the subepithelial stroma (**B**), and cell proliferation and collagen fibers were prominent in the subserosa (**C**), which were findings of chronic cholecystitis.

**Fig. 3:**
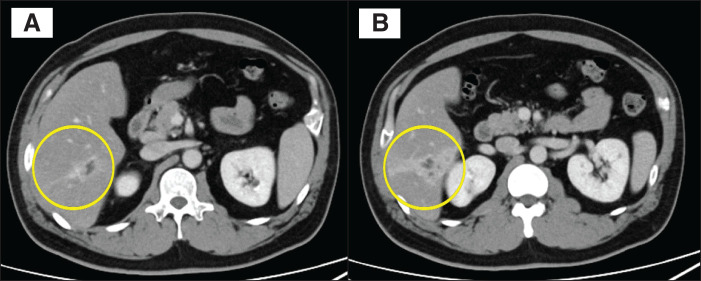
Computed tomography image (portal phase). A low-density area with contrast enhancement in the periportal wall and a filling defect are visible along the ventral region of segment 6; (**A**) is an axial slice on the cranial side and (**B**) is an axial slice on the caudal side.

**Fig. 4:**
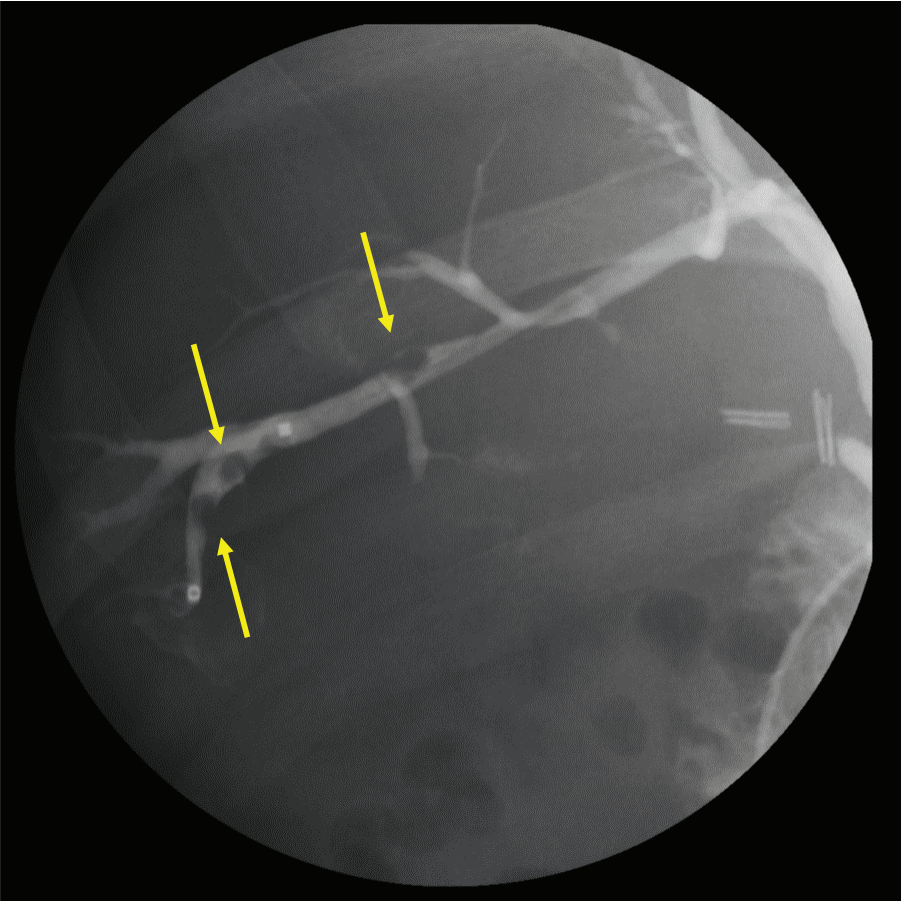
Endoscopic retrograde cholangiography image. Selective angiography of the ventral region of bile duct of segment 6 showing a filling defect due to multiple stones (yellow arrows).

**Fig. 5:**
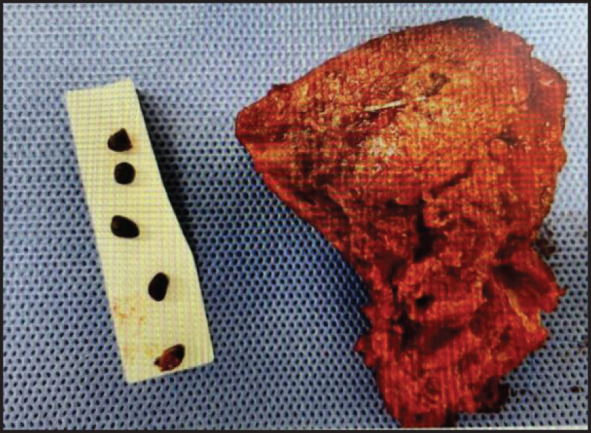
Surgical specimen. Hepatic segment 6 resection was performed. The background liver indicated fatty liver, and the resected liver weight was 130 g. Multiple stones were found in the bile duct of segment 6.

**Fig. 6:**
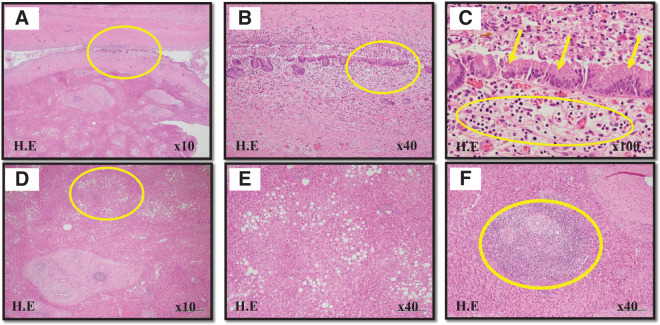
Microscopic images. (**A**) (original magnification ×10), (**B**) (original magnification ×40) and (**C**) (original magnification ×100) are hematoxylin-eosin-staining images of the bile duct. (**B**) shows that inflammatory cell infiltration is widespread in the bile duct. (**C**) shows swollen nuclei and roughened nuclear chromatin in the epithelial cells (yellow arrows), with inflammatory cell infiltration in the nearby stroma. (**D**) (original magnification ×10) and (**E**), (**F**) (original magnification ×40) are hematoxylin-eosin-staining images of the liver parenchyma. The background liver indicates fatty liver (**D** and **E**), with some inflammatory cell infiltration mainly composed of lymphocytes (**F**).

A 34-year-old man developed cholecystitis due to gallstones 8 years prior to presentation at our hospital and underwent laparoscopic cholecystectomy. At this time, intrahepatic stones were not detected in the preoperative evaluation (**Fig. 1**). Pathological examination shows chronic cholecystitis (**Fig. 2**). One year after the surgery, he developed cholangitis, and the following year he developed acute pancreatitis. Three years prior to presentation, he underwent endoscopic lithotripsy for common bile duct stones with cholangitis. At that time, intrahepatic lithiasis was detected in segment 6 (S6). However, there was no stenosis in the bile duct and no findings other than a dilated bile duct in S6 on imaging; therefore, the patient was kept under observation. During the observation period, he was subsequently admitted to the hospital with fever, abdominal pain, and elevated liver enzymes. The man's height was 1.67 m, his weight was 89 kg, and his BMI was 31.9. Physical examination revealed tenderness in the right hypochondrium. Blood laboratory testing revealed a serum aspartate aminotransferase concentration of 45 U/L, serum alanine transaminase concentration of 145 U/L, serum alkaline phosphatase concentration of 143 U/L, and serum C-reactive protein concentration of 66.6 mg/L. Enhanced computed tomography showed a low-density area in the ventral region of S6, dilatation of the bile duct with stones, and contrast effect on the periportal wall. The diagnosis was cholangitis due to intrahepatic stones (**Fig. 3**). Endoscopic retrograde cholangiopancreatography revealed intrahepatic lithiasis and a filling defect in the ventral region of bile duct of segment 6 (B6) (**Fig. 4**). An endoscopic nasobiliary drainage tube was placed at the periphery of B6 for drainage. There was no stenosis in B6, and no common bile duct stones were found. After drainage, the inflammatory response improved, and the dilatation findings had disappeared on repeat computed tomography. We then decided to perform laparoscopic hepatectomy of the ventral region of S6. The operation time was 363 min, the estimated blood loss was 100 ml, the weight of the resected liver was 130 g, and numerous stones were found in section B6 of the resected liver (**Fig. 5**). Pathological examination revealed bile duct inflammation and BilIN-1 in the bile duct epithelium, and atypical cells were present in the epithelial tissue just above the inflammatory cell infiltrate (**Figs. 6A–6C**); the bile duct resected margin was negative. The background liver indicates fatty liver, with some inflammatory cell infiltration (**Figs. 6D–6F**). The patient was discharged from the hospital on postoperative day 10. As of 2 years after surgery, no intrahepatic lithiasis or cholangitis has been seen.

## DISCUSSION

Biliary intraepithelial neoplasia is classified as BilIN and intraductal papillary neoplasia and is considered a precancerous lesion.^1–3)^ BilIN is classified as BilIn-1, BilIN-2, or BilIN-3 on the basis of the degree of nuclear dysmorphism.^4)^ BilIN-3 is an intraepithelial carcinoma that progresses from microinvasive to invasive carcinoma.^4,5)^ BilIN is a microscopic lesion that is almost detected in specimens of cholangiocarcinoma, and it is reported that BilIN was observed in 29.5%–58% of patients who underwent surgery for cholangiocarcinoma.^6,7)^ The incidence of cholangiocarcinoma varies by region worldwide, and Age-standardised incidence rates are in 3.1 to 3.5 cases per 100000 in Japan.^8)^ The peak age is between 60 and 70 years, and it is rarely diagnosed at a young age.^9)^ Regarding the pathophysiological mechanism, sustained chronic inflammation induces cytokine stimulation of the biliary epithelium, which engages various molecular pathways and induces neoplastic changes.^2,10)^ Possible etiologies include stones, primary sclerosing cholangitis, and common bile duct cysts,^4)^ all of which are thought to result from chronic inflammation.

Intrahepatic choledocholithiasis is defined as the presence of stones in the intrahepatic bile duct, and symptoms include fever, abdominal pain, and cholangitis with jaundice; however, there are also many asymptomatic courses.^11)^ Non-surgical treatments include endoscopic retrograde cholangiopancreatography, percutaneous transhepatic cholangioscopy, and oral administration of ursodeoxycholic acid.^11–13)^ Surgical treatment includes hepatectomy, with a low recurrence rate^14,15)^ and a carcinogenic rate of hepatolithiasis of 4.7%–6.8%.^16,17)^ Additionally, the stone recurrence rate within 5 years is 5.3% in hepatectomy cases.^18)^

In this case, patient was obese, as indicated by his body mass index, and a medical interview revealed that his eating habits were disordered. There was no specific family history, background disease, laboratory data, and there were no factors other than diet that led to intrahepatic lithiasis even after cholecystectomy. Pathological examinations of the hepatic specimen revealed that the inflamed area was widespread and BilIN was observed in some of the areas; therefore, inflammation was present independently, however, BilIN was not present independently. This reinforces the mechanism by which chronic inflammation causes cancer. It was thought that intrahepatic stones first formed and the bile duct thickened due to stimulation and inflammation, and that this repeated process led to the development of BilIN.

From the above, recurrent bile duct stones may be associated with precancerous lesions. Our patient had recurrent cholangitis due to intrahepatic stones at the same site, and hepatic resection was considered appropriate. Additionally, laparoscopic hepatic resection is noninferior to open hepatic resection regarding short- and long-term postoperative outcomes and complications, for segmental resection.^19,20)^

Surgical resection for intrahepatic stones in young patients should be considered because of the high incidence of malignancy and the low recurrence rate after surgical resection compared with endoscopic lithotripsy. Furthermore, minimally invasive laparoscopic hepatectomy is becoming safer to perform and should be performed in young patients with intrahepatic lithiasis.

## CONCLUSION

We reported a case of a young patient with recurrent intrahepatic lithiasis and cholangitis, diagnosed as BilIN after laparoscopic hepatectomy. In such a case, it is necessary to select a strategy that considers the coexistence of precancerous lesions, such as BilIN.

## ACKNOWLEDGMENTS

We thank Jane Charbonneau, DVM, from Edanz (https://jp.edanz.com/ac) for editing a draft of this manuscript.

## DECLARATIONS

### Funding

This research was supported by Grants-in-Aid for Scientific Research (KAKENHI: 24K19326 to J.Y. and 24K11898 to T.I.).

### Authors’ contributions

YY, JY, and TO developed the main concept and designed the study.

YY, JY, and TI drafted the manuscript.

All authors contributed to editing and critical revision of the manuscript for important intellectual content.

All authors were responsible for acquisition of the clinical data.

### Availability of data and materials

Not applicable.

### Ethics approval and consent to participate

The patient provided written consent to undergo the procedures described in the case report. This case report did not require approval from our institutional ethics committee.

### Consent for publication

Informed consent was obtained from the patient for publication of this case report.

### Competing interests

Authors declare no competing interests for this article.
